# Psychometric properties of the ORTO-R in a community-based sample of women and men from Poland

**DOI:** 10.1186/s40337-023-00734-x

**Published:** 2023-01-19

**Authors:** Anna Brytek-Matera, Sahar Obeid, Lorenzo Maria Donini, Marta Rogoza, Marta Marchlewska, Marta Plichta, Marzena Jezewska-Zychowicz, Souheil Hallit, Radosław Rogoza

**Affiliations:** 1 Institute of Psychology, University of Wrocław, Dawida 1, 50-527 Wrocław, Poland; 2 Social and Education Sciences Department, School of Arts and Sciences, Lebanese American University, Jbeil, Lebanon; 3 Department of Experimental Medicine, Sapienza University of Rome, Rome, Italy; 4 Institute of Psychology Polish Academy of Sciences, Warsaw, Poland; 5 Department of Food Market and Consumer Research, Institute of Human Nutrition Sciences, Warsaw University of Life Sciences (SGGW-WULS), Warsaw, Poland; 6 School of Medicine and Medical Sciences, Holy Spirit University of Kaslik, P.O. Box 446, Jounieh, Lebanon; 7 Research Department, Psychiatric Hospital of the Cross, Jal Eddib, Lebanon; 8 Applied Science Research Center, Applied Science Private University, Amman, Jordan; 9 Cardinal Stefan Wyszyński University in Warsaw, Warsaw, Poland; 10 Social Innovation Chair, University of Lleida, Lleida, Spain

**Keywords:** Orthorexia nervosa, ORTO-R, Polish sample

## Abstract

**Purpose:**

Over the past two decades, orthorexia nervosa (ON) has been increasingly investigated. Recently, a new revision version of the ORTO-15, namely ORTO-R, was used. The main objective of the present study was to confirm the factor structure of Polish version of the ORTO-R for evaluating ON thoughts and behaviors.

**Method:**

In three studies, a total of 3081 participants was selected by random sampling through several universities and companies. In Study 1, we used original items of ORTO-15, in Study 2 we used both, the ORTO-15 items and the revised items from ORTO-R, and in Study 3 we used only the items from the ORTO-R. Confirmatory factor analysis was used for determining the factorial structure of the Polish version of the ORTO-R. We also assessed internal consistency and convergent and criterion validity of the ORTO-R.

**Results:**

The model composed of the revised items (ORTO-R) was characterized of good convergent and criterion validity. Furthermore, ORTO-R appeared to be more internally consistent as compared to ORTO-15.

**Conclusion:**

The 6-item ORTO-R is valid and reliable method to assess orthorexic thoughts and behaviors among Polish-speaking population and could be applied in future research. Using revised version of the items is strongly preferred to using the items in their original ORTO-15 form.

## Plain English Summary

Over the past two decades, orthorexia nervosa (ON) has been increasingly investigated. Recently, a new revision version of the ORTO-15, namely ORTO-R, was used. We attempted in this paper to evaluate the psychometric properties of the Polish version of the ORTO-R. The Polish version of the ORTO-R is valid and internally consistent method to investigate ON and could be used in research and practices.

## Background

For over a decade, the most widely used self-report measure assessing orthorexia nervosa (ON) has been the ORTO-15 [1], however, its measurement quality is frequently questioned (e.g., unacceptable model fit, low internal reliability) [2, 3]. Two main limitations of the ORTO-15, which make researchers doubt in the accuracy of their results, are an inflated prevalence of ON and the unstable factorial structure of the ORTO-15 across different populations [4]. Although different national versions of ORTO-15 exists within the literature [3, 5–8], there is a high incongruence in terms of the number of factors and even the items retained in the final version of the measure. Nevertheless to this confusion, some items repeatedly appeared in all these different variations of the ORTO-15 [9]. On this basis, Rogoza and Donini [4] re-analyzed the factorial structure of the ORTO-15 using the original dataset [1] and proposed its revision, the ORTO-R. ORTO-R is composed of six items which the content was revised to better reflect ON thoughts and behaviors. Furthermore, the response scale has been changed to five-point Likert-type scale and the direction was reversed so the higher score reflects higher intensity of ON thoughts and behaviors. Also, given that the cut-off in ORTO-15 was frequently misused, ORTO-R was designed to assess orthorexic thoughts and behaviors in a dimensional manner, omitting the categorical diagnosis [4]. Finally, the order of items was changed to reduce the potential effects of the method bias.

To date, there are two studies within the literature on Lebanese population, which examined the ORTO-R. First study [10] used the items in their original form as they appeared in ORTO-15, without any modifications proposed in ORTO-R. Second study [10] examined the items in their revised version. While the former study struggled to provide adequate fit indices, results from the latter were better in terms of internal consistency and factorial structure. Furthermore, the study of Rogoza et al. [12] not only provided first to date evidence of ORTO-R factorial validity, but more importantly they provided robust evidence on the validity of the measurement in terms of assessment of its relations to other measure of ON thoughts and behaviors, eating disorders, perfectionism, depression, anxiety, and self-esteem. It appeared that ORTO-R is not only valid, but even outperforms the Teruel Orthorexia Scale in terms of the strength of relations to perfectionism and depression [11].

## Study objectives

The main objective of the present study was to evaluate whether the factor structure of Polish version of the ORTO-R will be replicated, and to assess its internal consistency and provide evidence of validity. In the first study, we used items in their original version taken out from the ORTO-15 [1]. In the second study, we used items from both, ORTO-15 and ORTO-R [12]. Finally, in Study 3 we used only the revised content of items. Accordingly to the results in the literature [10, 13], we expected that the revised items should outperform the former ones in terms of model fit to the data and internal consistency. In terms of convergent validity, in Study 1 we assessed its relations to symptoms and concerns characteristic of EDs (measured by EAT-26), in Study 2 we assessed the relations to eating behaviors and other orthorexic thoughts and behaviors. Finally, in the third Study we scrutinized the criterion validity testing if ORTO-R scores are related to health perceptions, and specific food behaviors (e.g., reading the list of ingredients) among general population. We expected that ON thoughts and behaviors should be positively related to eating disorders (Study 1). Furthermore, we expected that the ON thoughts and behaviors as measured by the ORTO-R should be positively related to different eating and orthorexic behaviors. Moreover, we expected that the ORTO-R, as compared to the items from ORTO-15 should be related more strongly to these variables (Study 2) Finally, we hypothesized that the ORTO-R scores should be positively related to one’s health perception as well as to worrying about health status as well as to specific food behaviors associated with ON (e.g., reading list of food ingredients; Study 3).

## Methods

### Participants and procedure

#### Study 1

A cross-sectional, population-based study was conducted in 1074 Polish adults were selected using a random sampling method. The study was conducted at several universities (from Silesia, Lower Silesia, Mazovia and Lesser Poland Province) and companies (volunteers from administrative and teaching personnel). Data of samples were collected via online survey. The notice about the research was distributed among social networking. The announcement included a link to study information, consent procedures (anonymous and voluntary nature of participation, freedom to refuse or withdraw without penalties) and the questionnaires. Informed consent was obtained from all participants before starting the survey (by ticking a respective box at the first page of the online survey) At any time and for any reason, they could refuse to answer a question or stop filling out the questionnaire and not send their data using the ‘send’ button. The participants received no financial incentive.

The study enrolled 1074 women (86.9%; *N* = 933) and men aged 18–45 years. The mean age of the sample was 23.42 years (SD = 3.80) and the mean Body Mass Index (BMI) was 21.59 kg/km^2^ (*SD* = 2.72). Of these participants, 10.42% (*N* = 112) presented a high level of disordered eating attitudes (a score at or above 20 on the Eating Attitudes Test-26).

#### Study 2

The sample included 478 students (87.9% women and 12.1% men). The average age of students was 22.30 (*SD* = 3.10). Most of the students were enrolled in bachelor and engineering degree studies (75.2%). The mean Body Mass Index (BMI) value was 22.30 (*SD* = 4.10 kg/m^2^). The students were not asked whether they had been diagnosed with a mental health disorder. Participants were recruited from eighteen universities, which were located in all macro-regions in Poland (northern, north-western, masovian, central, south-western, southern, eastern). The study was conducted using a computer-assisted web interview (CAWI) technique by Google form between March and November 2021. Students from health, nutrition and life sciences were invited to participate in the study. Health and nutrition fields were represented by 58.6% of students (food technology, human nutrition, dietetics, public health, technology and organization of gastronomy, chemical, and food analytics, and quality management and food analysis), 41.4% of participants were students of life science fields (biology, microbiology, chemistry, and biotechnology). The selection of participants in the second study was targeted, as dictated by the research findings, in which the risk of orthorexia nervosa was most often reported among those studying in nutrition and health-related fields. In addition, we have included students from the life sciences, because these students learn the fundamentals of biochemistry, analytical and organic chemistry, instrumental analysis techniques, and knowledge of key biological phenomena and processes in humans during their studies. This is information that nutrition and health students also learn. Therefore, these students have a similar core program. The study was conducted during lectures and workshops by an academic teacher. In addition, the invitation of the study was also posted on the universities’ websites, therefore, students visiting these websites could also participate in the study. Participants were informed about the purpose, course and duration of the study, and that they can opt out of the study at each stage without any consequences. Participation in the study was voluntary. Data confidentiality and anonymity were assured. The survey took approximately 20 min to complete. In total, 488 students participated in the study. Because of incomplete questionnaires, five students were excluded from the sample. Moreover, five students were excluded from the sample as they studied other sciences than those of health, nutrition and life (energetics, navigation and ship armament, finance and accounting and Polish philology). The final sample consisted of 478 students.

#### Study 3

A representative on gender, age and education population-based study was conducted in Polish adults. The study was conducted using a computer-assisted web interview (CAWI) technique by internal Polish research company in December 2021. Study was completely anonymous and voluntary nature. Participants had a freedom to refuse or withdraw from online survey without any penalties. In order to ensure engagement of respondents, three check questions were included in the survey where we asked respondents to tick a specific answer from a list. Respondents that failed more than one of these check questions were screened out from the survey. The final sample consisted of 1529 participants (51.9% women) aged between 18 and 83 (*M* = 47.14; *SD* = 15.89).

## Measures

### Study 1

#### Items from ORTO-15 retained in ORTO-R

We used the full ORTO-15 measure [1], however only six items which were identified as the best markers of ON were considered in the current study [4]. Each item is answered on a four-point Likert scale (1 = ‘‘always’’, 2 = “often”, 3 = “sometimes”, 4 = ‘‘never’’). Items receiving a score of 1 reflect an ON tendency, while those with a score of 4 points indicate normal eating habits. As ORTO-15 is a reverse-scoring test, therefore, lower scores indicates a more pathological behavior. In present study, we used the original Polish [7] version of the ORTO-15.

#### The Eating Attitudes Test (EAT-26)

The EAT-26 [14] is a widely used standardized self-report measure of symptoms and concerns characteristic of eating disorders (EDs). The EAT-26 test consists of 26 items to be scored on a 6-point scale (1 = “always”, 2 = “usually”, 3 = “often”, 4 = “sometimes”, 5 = rarely”, 6 = “never”). In the present research, we used a total score as an indicator of the eating disorder pathology. We used the original Polish version of the EAT-26 [15]. In the current sample, Cronbach’s α coefficients of the EAT-26 was 0.907.

### Study 2

#### Items from ORTO-15 retained in ORTO-R

##### ORTO-R

The ORTO-R [12] comprise same six items, the content of which was however revised (for a full English version of ORTO-R see: https://osf.io/8zv7q/). In addition to that, several amendments were included as compared to ORTO-15. First, the response scale has been changed to a five-point Likert-type scale (1 = “never”, 2 = “rarely”, 3 = “sometimes”, 4 = “often”, 5 = “always"). Second, the counter intuitive reverse-scoring has been changed so the higher the test score, the higher are the ON thoughts and behaviors. Third, the order of items has been randomly mixed so the items with the same beginning appeared in random order to reduce the method bias. In the current study, we used the Polish translation provided by the author of the scale, which, however, has been never subjected to any formal analysis. In Study 2, the order of the revised items was presented as they originally appeared in the ORTO-15, while in Study 3, the order of items was mixed as suggested by the authors [12].

##### Three-Factor Eating Questionnaire (TFEQ-13)

The TFEQ-13 is a self-assessment Polish version of a 13-items questionnaire used to measure eating behaviors [16, 17]. The TFEQ-13 includes three subscales: cognitive restraint (CR, 5 questions), uncontrolled eating (UE, 5 questions), and emotional eating (EE, 3 questions). Twelve questions were scored on a four-point Likert scale: 0 = “definitely not”, 1 = “rather not”, 2 = “rather yes”, and 3 = “definitely yes”. The question thirteen was scored on an eight-point Likert scale ranging from 1 = “I never restrain from eating” to 8 = “I always restrain from eating”, and was recoded as follows: 1 or 2 (into 0), 3 or 4 (1), 5 or 6 (2), and 7 or 8 (3). In the present study, in the internal consistency estimates were: α = 0.77 for cognitive restraint, α = 0.76 for uncontrolled eating, and α = 0.86 for emotional eating.

##### The Düsseldorf Orthorexia Scale (PL-DOS)

The PL-DOS is a self-report Polish version of a questionnaire for assessing and screening ON [18]. The scale consists of 10 items with the following answer options: 1 = “this does not apply to me”, 2 = “this does rather not apply to me”, 3 = “this does somewhat apply to me”, and 4 = “this applies to me”. In this study, the DOS-PL has shown high internal consistency (α = 0.80).

### Study 3

#### ORTO-R

##### Health perception

Health perception was measured by using two questions: (1) “To what degree are you worried about your health?” where answers were scored on a five-point Likert scale: 1 = “not at all”, 2 = ”a little”, 3 = “a moderate amount”, 4 = “a lot”, 5 = “a great deal”, (2) “How is your health in general?” where answers were also scored on a five-point Likert scale: 1 = “poor”, 2 = “fair”, 3 = “good”, 4 = “very good”, 5 = “excellent”.

##### Conscious Food Choices

The Conscious Food Choices Scale is a self-report Polish version of a three-items questionnaire used to measure conscious consumption [19], i.e. “Before buying a food product, I will read the nutrition information displayed on the label”, “Before buying a food product, I will pay attention to the country of origin of the groceries that I will be buying”, “Before buying a food product, I will pay attention to how much the food products are processed”. Participants were asked to determine their willingness to do these things on a scale from 1 = “I definitely will not do this” to 5 = “I will definitely do this”.

### Statistical analyses

To test the internal structure of the ORTO questionnaires, a confirmatory factor analysis (CFA) was carried out in Mplus v. 7.2. [20], using robust maximum likelihood estimation to account with the lack of multivariate normality. Missing observations were handled using the full information maximum likelihood estimation. We assessed a model in which a single latent variable was loaded by six indicators. To evaluate the goodness-of-fit of a model, multiple indices were calculated; the root mean square error of approximation (RMSEA) (values ≤ 0.06 indicate a good-fitting model and values > 0.10 indicate poor model), the comparative fit index (CFI) and Tucker-Lewis Index (TLI) (values ≥ 0.90 indicate good model fit), and standardized root mean square residual (SRMR) (values < 0.05 show good fitting models) [21, 22]. Internal consistency of the ORTO-R was calculated using Cronbach’s α values. To assess validity, we used the Pearson’s correlation test. To compare whether the correlations between items from ORTO-15 and ORTO-R are different (Study 2), *Z*-test was applied.

## Results

### Factorial structure of the ORTO-R using original vs revised items

The model fit to the data using the items from the ORTO-15 was fitted poorly in Study 1 (χ_(9)_^2^ = 616.16; *p* < .001; CFI = .469; TLI = .115; RMSEA = .251 [.234, .268]; SRMR = .145) and suboptimal in Study 2 (χ_(9)_^2^ = 36.48; *p* < .001; CFI = .897; TLI = .829; RMSEA = .080 [.054, .108]; SRMR = .045). The ORTO-R was fitted poorly in Study 2 (χ_(9)_^2^ = 181.56; *p* < .001; CFI = .643; TLI = .405; RMSEA = .200 [.175, .226]; SRMR = .088) and suboptimla in Study 3 (χ_(9)_^2^ = 173.87; *p* < .001; CFI = .923; TLI = .871; RMSEA = .109 [.096, .124]; SRMR = .050). Inspection of the modification indices revealed an extreme collinearity of a pair of residuals in Study 1 (i.e., residuals of items 3 and 7 as they appeared in ORTO-15; MI = 714.06). The correlation between same ORTO-15 items in Study 2 was negligible (MI = 12.79). For ORTO-R, in Study 2 and 3, a remarkable correlation between these residuals appeared as well, albeit it was of less magnitude as compared to Study 1 (MI = 125.31; MI = 139.10, respectively). Accounting for this covariation between residuals in the model significantly improved model fit in all studies, which was now: χ_(8)_^2^ = 29.63; *p* < .001; CFI = .981; TLI = .965; RMSEA = .050 [.032, .070]; SRMR = .028 for ORTO-15 in Study 1 and χ_(8)_^2^ = 24.17; *p* = .002; CFI = .939; TLI = .886; RMSEA = .065 [.036, .096]; SRMR = .034 for ORTO-15 in Study 2; χ_(8)_^2^ = 40.70; *p* < .001; CFI = .932; TLI = .873; RMSEA = .092 [.065, .122]; SRMR = .051 for ORTO-R in Study 2, and χ_(8)_^2^ = 33.32; *p* < .001; CFI = .988; TLI = .978; RMSEA = .045 [.030, .062]; SRMR = .018 for ORTO-R in Study 3. The inclusion of the covariation in residuals was similar for both analyzed models, with slight preference to the ORTO-R. The standardized factor loadings are given in Table 1. The strength of the factor loadings was apparently higher for ORTO-R (all > .40; except for the item 5 in Study 2) as compared to ORTO-15 (with two loadings ≤ .30). Summing up, the structural analyses supported both, the ORTO-15 and ORTO-R, however, the results favors ORTO-R over the ORTO-15 in terms of how the former was fitted to the data.

**Table 1:** Standardized factor loadings of the ORTO-15 and ORTO-R measurement models

ORTO-15 content	ORTO-R content	ORTO-15 Study 1	ORTO-15 Study 2	ORTO-R Study 2	ORTO-R Study 3
3. In the last 3 months, did the thoughts of food worry you?	4. In the last three months, did the thoughts of food make you feel guilt, ashamed, and anxious?	0.24	0.30	0.38	0.49
4. Are your eating choices conditioned by your worry about your health status?	1. Are your rigid and restrictive dietary choices conditioned by your worry about your health status?	0.47	0.47	0.50	0.64
7. Does the thought about food worry you for more than three hours a day?	5. Does thinking about food excessively worry you for more than three hours a day?	0.22	0.14	0.26	0.45
10. Do you think that the conviction to eat only healthy food increases self-esteem?	2. Would you agree that eating healthy food increases self-esteem?	0.66	0.68	0.81	0.80
11. Do you think that eating healthy food changes your lifestyle (frequency of eating out, friends, …)?	6. Does eating healthy food changes your lifestyle (frequency of eating out, friends, …)?	0.62	0.51	0.49	0.71
12. Do you think that consuming healthy food may improve your appearance?	3. Do you believe that strict consuming only of healthy food may improve your appearance?	0.60	0.60	0.58	0.74
Covariation between residuals of items (3 and 7 in ORTO-15, 4 and 5 in ORTO-R)		0.64	0.17	0.50	0.35

### Internal consistency

The internal consistency for ORTO-15 items equaled α = 0.67 in Study 1 and α = 0.61 in Study 2. The item-total correlations ranged between .32 to .46 in Study 1 and .17 to .46 in Study 2. The estimate for ORTO-R equaled α = 0.71 in Study 2 and α = 0.82 in Study 3. The item-total correlations ranged between .35 to .55 in Study 2 and .48 to .67 in Study 3. Thus, the obtained results clearly provides more support for the ORTO-R, which is much more internally consistent measure than its ancestor measure.

### Validity of the ORTO-R

Table 2 presents relations of the ORTO-R to EAT-26 scores (Study 1), eating and orthorexic behaviors (Study 2) and perceived health status, worrying about health, and specific food behaviors (Study 3). Expectedly, ON thoughts and behaviors were positively related to the EAT-26 scores. The ON thoughts and behaviors as assessed by ORTO-R were related to all eating behaviors, and especially to uncontrolled eating and strongly related to orthorexic behaviors as measured by the DOS. Comparing the correlations to criterion validity measures between ORTO-R and ORTO-15, the ORTO-R outperformed ORTO-15 (i.e., was related more strongly) in regard to all scales. Although the perceived health status and worrying about health were negatively related one to another, they were both related positively to ORTO-R scores.^1^ Finally, the ORTO-R scores were positively related to all specific food behaviors associated with the ON such as reading the list of ingredients. These results supports the validity of the ORTO-R measurement.

**Table 2:** Relations of ORTO scores to criterion validity variables

	1				
Study 1					
1 ORTO-15	–				
2 EAT-26*	− 0.52***				

	1	2	3	4	5
Study 2					
1 ORTO-R	–				
2 ORTO-15	− 0.63***				
3 Cognitive restraint	0.19***	− 0.07			
4 Uncontrolled eating	0.46***	− 0.36***	− 0.03		
5 Emotional eating	0.17***	− 0.07	0.45***	0.14**	
6 DOS	0.66***	− 0.51***	0.07	0.55***	0.14**

	1	2	3	4	5
Study 3					
1 ORTO-R	–				
2 Health status	0.07**	–			
3 Worrying about health	0.17***	− 0.41***			
4 Reading ingredients	0.35***	0.01	0.10***		
5 Reading country of origin	0.26***	− 0.01	0.12***	0.65***	
6 Food processing	0.37***	0.03	0.10***	0.78***	0.68***

Negative correlation between the items from the ORTO-15 to EAT-26 in Study 1 and other scales in Study 2 results from the reverse coding in ORTO-15 administered in Study 1 and 2. This result should be interpreted that ON thoughts and behaviors are positively associated to EAT-26 and other scores

*p < .05; **p < .01; ***p < .001

## Discussion

The main objective of the present study was to investigate the psychometric properties of the Polish version of the ORTO-R in a general population sample. For this purpose, we conducted three studies. We compared the extent to which the revised ORTO-R items works in terms of factorial validity, convergent and criterion validity, and internal consistency as compared to the unchanged items from the ORTO-15. The CFA provided support for both versions, however, we identified a large covariation between residuals between a pair of items regarding issues of affectivity. In unmodified ORTO-15 version, not accounting for this covariation led to by far unacceptable fit indices (at least in Study 1), while in ORTO-R this effect has been largely diminished through modification of the measure. Furthermore, the factor loadings in ORTO-R were much stronger than in ORTO-15. These results are congruent with two studies on Lebanese populations, which assessed either items from ORTO-15 or ORTO-R [10, 13]. The estimates of internal consistency were acceptable for both versions, however they reached much more preferable estimates for the revised version of the measure. Thus, our results supports notion of the Rogoza and Donini [4] that the revision of the ORTO measure is a necessary step forward. Finally, our results not only supported the internal properties of the measure, but also provided support for its validity. In regard to convergent validity, ORTO-R was more strongly related to different eating behaviors as well as to other measure of orthorexic thoughts and behaviors than was the ORTO-15. We have demonstrated evidence of criterion validity providing evidence that the ORTO-R scores are associated not only with a positive health perception, but it is simultaneously related to worrying about one’s health. This result is especially interesting as these variables were in fact negatively related one to another. This might emphasize the fact that while ON is indeed focused on healthiness, it is also underpinned by being worried [23]. While ORTO-R has been revealed to be positively related to other measures of ON [10], we also demonstrated that it is related to specific food behaviors such as reading the food ingredients. Summing up, the gathered evidence supports the validity of the Polish adaptation of the ORTO-R.

Our study had some limitations. Firstly, the conducted studies were cross-sectional and therefore one cannot conclude about causal relationship; the cause-effect relationship is a limitation of our study only within the context of criterion validity of the Polish version of the ORTO-R. Secondly, the divergent validity and test–retest reliability of the ORTO-R were not examined, both of which would provide a more complete view of ORTO-R’s psychometric properties. Third, within the student samples there were an underrepresentation of males which could also impact our results. Finally, the clinical interview for assessing eating disorders and other mental disorder was not used in the present study.

## Conclusion

Our findings confirmed the factor structure of the Polish version of the ORTO-R, and provided evidence of factorial, convergent, and criterion validity in three large community-based samples from Poland.

The Polish version of the ORTO-R is valid and internally consistent method to investigate ON and could be used in research and practices. This study demonstrates that it is insufficient to use items from the ORTO-15 and the revised content from ORTO-R should be preferred.

## Data Availability

The dataset used during the current study are available from the corresponding author on reasonable request.

## Footnotes

1 The strength of these estimates was even stronger when accounting for their shared variance within a linear regression model (β = .16 for perception of health status, and β = .23 for worrying about health).
